# Innovative microbial disease biocontrol strategies mediated by quorum quenching and their multifaceted applications: A review

**DOI:** 10.3389/fpls.2022.1063393

**Published:** 2023-01-12

**Authors:** Xixian Zhu, Wen-Juan Chen, Kalpana Bhatt, Zhe Zhou, Yaohua Huang, Lian-Hui Zhang, Shaohua Chen, Junxia Wang

**Affiliations:** ^1^ State Key Laboratory for Conservation and Utilization of Subtropical Agro-bioresources, Guangdong Province Key Laboratory of Microbial Signals and Disease Control, Integrative Microbiology Research Centre, South China Agricultural University, Guangzhou, China; ^2^ Guangdong Laboratory for Lingnan Modern Agriculture, College of Plant Protection, South China Agricultural University, Guangzhou, China; ^3^ Department of Food Science, Purdue University, West Lafayette, IN, United States

**Keywords:** quorum quenching, quorum sensing, biocontrol, acyl homoserine lactones, diffusible signal factor

## Abstract

With the increasing resistance exhibited by undesirable bacteria to traditional antibiotics, the need to discover alternative (or, at least, supplementary) treatments to combat chemically resistant bacteria is becoming urgent. Quorum sensing (QS) refers to a novel bacterial communication system for monitoring cell density and regulation of a network of gene expression that is mediated by a group of signaling molecules called autoinducers (AIs). QS-regulated multicellular behaviors include biofilm formation, horizontal gene transfer, and antibiotic synthesis, which are demonstrating increasing pathogenicity to plants and aquacultural animals as well as contamination of wastewater treatment devices. To inhibit QS-regulated microbial behaviors, the strategy of quorum quenching (QQ) has been developed. Different quorum quenchers interfere with QS through different mechanisms, such as competitively inhibiting AI perception (e.g., by QS inhibitors) and AI degradation (e.g., by QQ enzymes). In this review, we first introduce different signaling molecules, including diffusible signal factor (DSF) and acyl homoserine lactones (AHLs) for Gram-negative bacteria, AIPs for Gram-positive bacteria, and AI-2 for interspecies communication, thus demonstrating the mode of action of the QS system. We next exemplify the QQ mechanisms of various quorum quenchers, such as chemical QS inhibitors, and the physical/enzymatic degradation of QS signals. We devote special attention to AHL-degrading enzymes, which are categorized in detail according to their diverse catalytic mechanisms and enzymatic properties. In the final part, the applications and advantages of quorum quenchers (especially QQ enzymes and bacteria) are summarized in the context of agricultural/aquacultural pathogen biocontrol, membrane bioreactors for wastewater treatment, and the attenuation of human pathogenic bacteria. Taken together, we present the state-of-the-art in research considering QS and QQ, providing theoretical evidence and support for wider application of this promising environmentally friendly biocontrol strategy.

## 1 Introduction

Quorum sensing (QS) is an effective mechanism, by which various bacteria can regulate their gene expression accordingly and synchronize their biological behaviors based on their population density (Fuqua et al., 1994; Wang et al., 2020). In a QS system, bacteria synthesize, secrete, and sense QS signaling molecules, which are also called auto-inducers (AIs). QS was first reported in the 1970s (Nealson et al., 1970), through which the marine bacterium *Vibrio fischeri* can regulate its biological luminescence. Since then, studies on QS have promoted the discovery of diverse QS signals and QS-regulated traits in a broad range of bacteria and archaea, and even in eukaryotes (Grandclément et al., 2016). QS is closely related to many microbial biological behaviors, including bioluminescence, biofilm formation, antibiotic synthesis, mobility, sporulation, and gene exchange (Nealson et al., 1970; Davies et al., 1998; Bhatt et al., 2022; Mishra et al., 2022).

In terms of their molecular structure, QS signaling molecules can be briefly categorized into three groups: (1) The QS signaling molecules in Gram-negative bacteria are mostly derivatives of fatty acids, such as *N*-acylhomoserine lactones (AHLs) and *cis*-11-methyl-2-dodecenoic acid (DSF); (2) the signaling molecules in Gram-positive bacteria are mostly oligopeptides, such as autoinducing peptides (AIPs); and (3) other signaling molecules, including autoinducer-2 (AI-2), *Pseudomonas* quinolone signal (PQS), integrated quorum-sensing signal (IQS), 3-hydroxy-methyl palmitate (3-OH-PAME), dialkyl resorcinols (DARs), α-pyrone, p-coumaroyl-HSL (aryl-HSL) (Shi, 2021; Brameyer et al., 2015a, b; Xu and Liu, 2011), and so on.

In the past few decades, antibiotics have been widely applied in various fields to kill the plant and human pathogens, resulting in increased pathogen resistance and decreased effectiveness of such chemicals. However, contrary to the trend of rising chemical resistance, the invention of new drugs has dramatically declined over the last several decades (Bhatt et al., 2020; Zhao, 2020), highlighting the necessity of discovering and/or inventing new anti-pathogen drugs.

Quorum quenching (QQ) refers to all processes involved in the disturbance of QS (Grandclément et al., 2016; Zhang et al., 2020), which has shown great advantages over traditional anti-bacterial strategies, due to its low probability of generating bacterial resistance (Wang H et al., 2022). Quorum quenchers can be divided into two categories: QQ enzymes, which inactivate QS signals, and QS inhibitors (QSIs), which chemically disrupt QS pathways (e.g., competitive inhibition of signal receptors). For example, an AHL-degradation enzyme purified from *Rhodosporidium toruloides* could inactivate a broad range of AHLs, from short-chain (C4-HSL) to long-chain ones (C14-HSL) (Luo, 2010). In another study, *in silico*, and *in vitro* tests demonstrated that a natural plant compound, phytol, bound to AHL receptors of *Chromobacterium violaceum* with high affinity and effectively reduced QS-regulated behaviors (e.g., biofilm formation, cell aggregation, and alkaline protease activity) Vargas et al., 2021. Aside from the QSIs and QQ enzymes, quorum quenching can also occur under purely physical conditions; for example, through lactonolysis of AHL compounds—the opening of the lactone ring with the addition of H_2_O occurs spontaneously in aqueous solutions (Grandclément et al., 2016).

In this review, we summarize decades of studies in quorum sensing and quorum quenching, highlighting the multi-faceted applications of quorum quenchers. In the first section, the significant role of quorum sensing in regulating biological behaviors is introduced. Next, we describe the various signaling molecules employed by various micro-organisms, as well as the action mode of the QS system. Moreover, QSIs and QQ enzymes are categorized in detail, according to different derivations or QQ mechanisms. Finally, applications of quorum quenching are presented in the context of different fields, including agriculture, aquaculture, and waste treatment, emphasizing the profound advantages of QQ enzymes and QQ bacteria, when compared to traditional strategies.

## 2 Quorum sensing

**Figure 1:**
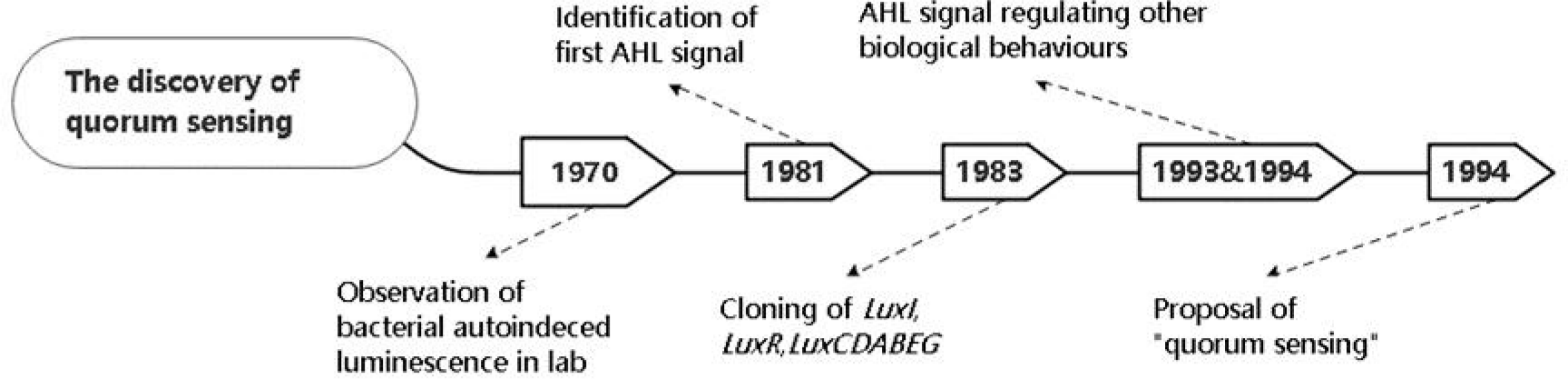
The timeline shows the brief history of the discovery of quorum sensing.

Quorum sensing (QS) is a sophisticated molecular mechanism, by which microorganisms sense their overall population density, allowing them to trigger the expression of target genes and synchronize their behavior (Fan et al., 2017). In the study of the bioluminescence of *Photobacterium fischeri* in 1970, scientists observed that luciferase is synthesized massively in a relatively short burst during the period of exponential growth, while it is inactive after transferring to a freshly inoculated culture (Nealson et al., 1970). The autoinducer excreted from *Photobacterium fischeri* was isolated and identified as *N*-(3-oxohexanoyl)-3-aminodihydro-2(3H)-furanone (Eberhard et al., 1981). In 1983, Engebrechte et al. (1983) cloned and identified *LuxI* (autoinducer synthetase gene), *LuxR* (autoinducer receptor gene) and *LuxCDABEG* (luciferase-generated genes clusters). In the 1990s, a diffusible conjugation factor (CF) was considered as an enhancer of Ti plasmid conjugal transfer in *A. tumefaciens* (Zhang and Kerr, 1991), and it was later identified as a member of AHL family with a similar structure to bioluminescence autoinducer of *Vibrio fischeri* (Zhang et al., 1993), suggesting AHLs are part of a variety of conserved signals involved in bacterial gene regulation. Subsequently, members of AHL family were identified in the regulation of virulence gene expression in *Erwinia carotovora* and *Pseudomonas aeruginosa* (Jones et al., 1993; Pearson et al., 1994). In a review written by Fuqua et al. (1994) “quorum sensing” was first proposed as a universal mechanism regulating population behaviors in various bacterial species. The brief history of QS discovery is depicted in **Figure 1**.

Until now, scientists have demonstrated that various group behaviors are correlated with QS, such as swarming motility, biofilm formation, expression of secretion systems, and the production of antibiotics, exopolysaccharides, elastase, protease, hemolysin, and rhamnolipids (Salmond et al., 1995; Uroz and Oger, 2017; Bzdrenga et al., 2017; Bhatt et al., 2022). In the pathogenic cycle, biofilm formation not only serves as important means for bacteria to cope with stress but also serves as an important pathogenic factor (Song et al., 2017). In many bacterial pathogens, QS is involved in the switching between commensal or saprophytic lifestyles to pathogenic cycles (Fan et al., 2017). This is exactly the case for *Pseudomonas aeruginosa*, an opportunistic human pathogen that can proliferate and accumulate QS signals in wounds; as a result, the expression of virulence factors is triggered (Fan et al., 2017). In another case, PhcB in *Ralstonia solanacearum* (a phytopathogenic bacterium) might be a small-molecule *S*-adenosylmethionine-dependent methyltransferase, which catalyzes the synthesis of 3-OH-PAME from a natural fatty acid. 3-OH-PAME stimulates the expression of *eps* (the biosynthetic locus for extracellular polysaccharide) and was considered as part of a new family of compounds in QS systems (Flavier et al., 1997).

## 3 QS signaling molecules and QS system

In this section, we categorize the different QS signaling molecules employed by different micro-organisms and demonstrate the action mode of QS systems.

### 3.1 AHLs and LuxI/LuxR system

Acyl homoserine lactones (AHLs), also known as autoinducer-1 (AI-1) (Bzdrenga et al., 2017), are the most widely existing and intensively studied QS signaling molecules (Deng et al., 2013). The chemical structure of AHL is composed of a lactone ring linked with an acyl chain by an amide bond. AHLs differ in their acyl side-chain, which usually contains 4 to 18 carbons with oxygen or hydroxyl substitution at the third carbon (Uroz and Oger, 2017; Du, 2021). This principle also provides nomenclature; for example, *N*-(hexanoyl)-*L*-homoserine lactone will appear as C6-HSL, while *N*-(3-hydroxyoctanoyl)-*L*-homoserine lactone can be abbreviated as OHC8-HSL (Grandclément et al., 2016).

In 1994, the mechanism of the QS-regulated LuxI/LuxR system responsible for bioluminescence was demonstrated (Fuqua et al., 1994). It was found that LuxI encodes the synthase of a signaling molecule, 3-oxo-C6-HSL (OC6-HSL) which, in turn, activates the expression of the LuxI gene cluster by binding to the LuxR protein (Fuqua et al., 1994). When the cell density is low, LuxI and LuxR are expressed constitutively but at low concentrations, and AHLs passively diffuse out of cells down the concentration gradient (Fuqua et al., 1994). As the concentration of AHL increases, in proportion to cell density, its intracellular concentration reaches a threshold that allows for specifically binding to LuxR, thus triggering the mass expression of target genes (Fuqua et al., 1994; Du, 2021).

After the landmark works of Nealson et al. (1970) and Fuqua et al. (1994), further studies have demonstrated the functional mechanisms of LuxI and LuxR. Evidence has shown that LuxI catalyzes the synthesis of AHL through S-adenosylmethionine (SAM) and ACP (Parsek et al., 1999). LuxR, which contains a ligand-binding domain on the N-terminal and a DNA-binding domain on the C-terminal, functions as both an AHL receptor and transcription regulation factor. Notably, some bacteria possess LuxR receptors, yet lack any LuxI-type synthase; these are, thus referred to as LuxR orphans or solos (Brameyer et al., 2015a). This is exactly the case for *Escherichia coli*, *Salmonella enterica*, and *Photorhabdus asymbiotica*.

More than two hundred bacteria was reported to produce signal factor of AHL family (Zhang, 2019), among which the majority utilize LuxI for its synthesis, while the rest use LuxM, AinS, VanM, or HtdS (Luo, 2010), among others. For example, *Acinetobacter baumannii* uses AbaI/AbaR as a homologue to the LuxI/LuxR system, to synthesize and sense AHLs (Dong et al., 2021). *Methanosaeta harundinacea* 6Ac is a methanogenic archaeon encoding a FilI protein responsible for the synthesis of carboxylated AHLs. FilI, a LuxI ortholog, can also be identified in other methanogenic genomes (Zhang et al., 2012).

The AHL-mediated system mediates diverse biological behaviors in microbes, including bioluminescence (Fuqua et al., 1994; Salmond et al., 1995), Ti plasmid conjugal transfer (Zhang et al., 2002), production of cell-wall degrading enzymes (Loh et al., 2002), type VI secretion system (Khajanchi et al., 2009), biofilm formation (Khajanchi et al., 2009), and protease production (Khajanchi et al., 2009).

### 3.2 Autoinducer-2

Autoinducer-2 (AI-2) refers to a group of molecules that are in equilibrium with each other and their precursor, 4,5-dihydroxy-2,3-pentanedione (DPD).

Unlike many quorum-sensing signaling molecules (e.g., AHLs) produced only by a particular species or a narrow range of closely related species (Defoirdt, 2018), AI-2 is conserved in several Gram-positive and Gram-negative bacteria (Zhao, 2020) and considered to be an inter-species communication signal among bacteria (Rao, 2021). AI-2 has been detected in pathogens such as *Salmonella typhimurium* and *V. cholerae*. Interestingly, AI-2 has also been detected in bacterial cultures lacking either a QS-like response to the signal or the *LuxS* gene (i.e., AI-2 synthase). Some authors have, therefore, proposed that it could be a universal metabolic by-product, rather than a QS signal (Grandclément et al., 2016).

### 3.3 DSF and RpfC/RpfG system

Diffusible signal factor (DSF) is a type of cis unsaturated fatty acid, with the chemical structure of *cis*-11-methyl-2-dodecylene acid (Ye et al., 2020a; Deng et al., 2015, 2016). Barber et al. (1997) have reported a model for the DSF system in *Xanthomonas campestris* pv. *campestris* (*Xcc*), a novel mechanism for regulating virulence factor synthesis (Barber et al., 1997). In fact, DSF and its homologs, such as *cis*-2-dodecenoic acid (BDSF), *cis*,*cis*-11-methyldodeca-2,5-dienoic acid (CDSF), and *cis*-10-methyl-2-dodecenoic acid (IDSF), are widely conserved in a variety of Gram-negative pathogens, including *Xcc* and *Xanthomonas oryzae* pv. *oryzae* (*Xoo*) (Deng et al., 2014; Ye et al., 2020b; Rao, 2021; Song et al., 2021).

The DSF-mediated QS system is composed of DSF synthase RpfF and regulation factor RpfC/RpfG (Rao, 2021). DSF activates RpfC for autophosphorylation and transports the phosphate group to RpfG, which can activate the catalytic activity of phosphodiesterase, thus down-regulating the concentration of c-di-GMP. Eventually, the concentration of Clp (CAP-like protein) increases, directly or indirectly regulating the expression of nearly 300 genes (He et al., 2007; He and Zhang, 2008; Tao et al., 2010; Rao, 2021).

### 3.4 Autoinducing peptides

Autoinducing peptides (AIPs), also referred to as peptide-pheromones, are mainly used by Gram-positive bacteria, and are typically species- or strain-specific (Fan et al., 2017). There are two forms of AIPs: there are cyclic AIPs, which have been detected in *Staphylococcus aureus*, while *Streptococcus pneumoniae* and *Bacillus subtilis* use linear AIPs (Rao, 2021). Unlike other signaling molecules, which diffuse through the cell membrane, AIPs are transported to extracellular space by the ABC transport system (Rao, 2021). AIPs are encoded by the *agrD* as propeptides, and are exported by the trans-membrane protein AgrB. At high population density, AIPs are sensed by a two-component system, which consists of a transmembrane receptor AgrC and a response regulator AgrA.

### 3.5 Other signaling molecules

In addition to the above-mentioned QS signaling molecules, some bacteria utilize other signaling molecules to mediate their QS system (Rao, 2021). In this section, we describe several less-reported signaling molecules.

AI-3 is a rarely reported signal, which is considered to be derived from tyrosine. It can be produced by the intestinal flora and is received by the QseC receptor (Zhao, 2020). Diketopiperazines (DKPs) are found in *Pseudomonas fluorescens*, *Pseudomonas alcaligenes*, *Enterobacter agglomerans*, and *Citrobacter freundii* (Luo, 2010). 3-Hydroxypalmitate methyl ester (3-OH-PAME) is utilized by *Ralstonia solanacearum* as a QS signal, with PhcS-PhcR as the regulation system (Rao, 2021). Dialkylresorcinols (DARs) and cyclohexanediones (CHDs) have been identified as QS signals in *Photorhabdus asymbiotica*, an insect and human pathogen (Brameyer et al., 2015a). These two molecules are synthesized by Pcf in the DarABC synthesis pathway, and are sensed by the LuxR homolog PauR (Brameyer et al., 2015a).

### 3.6 Co-existing multiple QS systems regulated by different QS signals

Numerous Gram-negative bacteria utilize more than one QS system, and may combine these systems either in an additive model (Luo, 2010; Bar-Rogovsky et al., 2013) with distinct or partially overlapping systems (Zhang et al., 2012), or as a hierarchical model, in which one system induces a second one (Eberhard et al., 1981). On one hand, some bacteria utilize different systems to synthesize and sense one signaling molecule; for example, *Erwinia carotovora* use CarI/CarR and ExpI/ExpR simultaneously to synthesize and sense OHHL (OC_6_-HSL) (Luo, 2010). On the other hand, some bacteria integrate multiple signal inputs for communication; for example, the QS system of the *P. aeruginosa* complex regulatory network consists of four sub-units, LasI/LasR, RhlI/RhlR, pqs, and iqs, which each use one signal: N-oxododecanoyl-L-homoserine lactone (OdDHL), N-butanoyl-L-homoserine lactone (BHL), Pseudomonas quinolone signal (PQS), and the Integrated quorum sensing signal (IQS), respectively (Uroz and Oger, 2017). The four sub-units are organized hierarchically, with the LasI/LasR system at the top of the hierarchy (Uroz and Oger, 2017). In another case, *Burkholderia plantarii* possesses two QS systems: One is the AHL-mediated system, that synthesizes AHL by PlaI which is received by PlaR; the other is the DSF-mediated system, which uses RpfF as a synthase and RpfC/RpfG or RpfR as receptors (Haruna et al., 2016).

## 4 Quorum quenching strategy

Many pathogens regulate the expression of virulence factors through the QS system (Yu et al., 2018); therefore, the antibacterial strategy of blocking QS has attracted more and more attention as a novel strategy against bacterial diseases (Yu et al., 2018). Unlike drug resistance generated by traditional antibiotics, quorum quenchers generate resistance in mutants called QS-non-responsive phenotype (Defoirdt, 2018); however, as quorum quenchers impose no direct selective pressure on bacteria, it is considered that resistance to quorum quenchers is far less common, compared to antibiotic resistance.

Quorum quenching (QQ) refers to mechanisms that interfere with quorum sensing systems, inhibiting the expression of QS-mediated genes and the appearance of QS-regulated traits through the utilization of quorum quenchers (e.g., QQ enzymes or QSIs) (Song et al., 2017). This strategy was first described in 2000, with the identification of an AHL-inactivating enzyme, AiiA, in *Bacillus* sp. 240B1 (Dong, 2000). The expression of *aiiA* in the transformed *Erwinia carotovora* strain SCG1 significantly decreased extracellular pectolytic enzyme activities, and attenuated its pathogenicity by reducing the release of AI (Dong, 2000).

### 4.1 Mechanisms of quorum quenching

**Figure 2:**
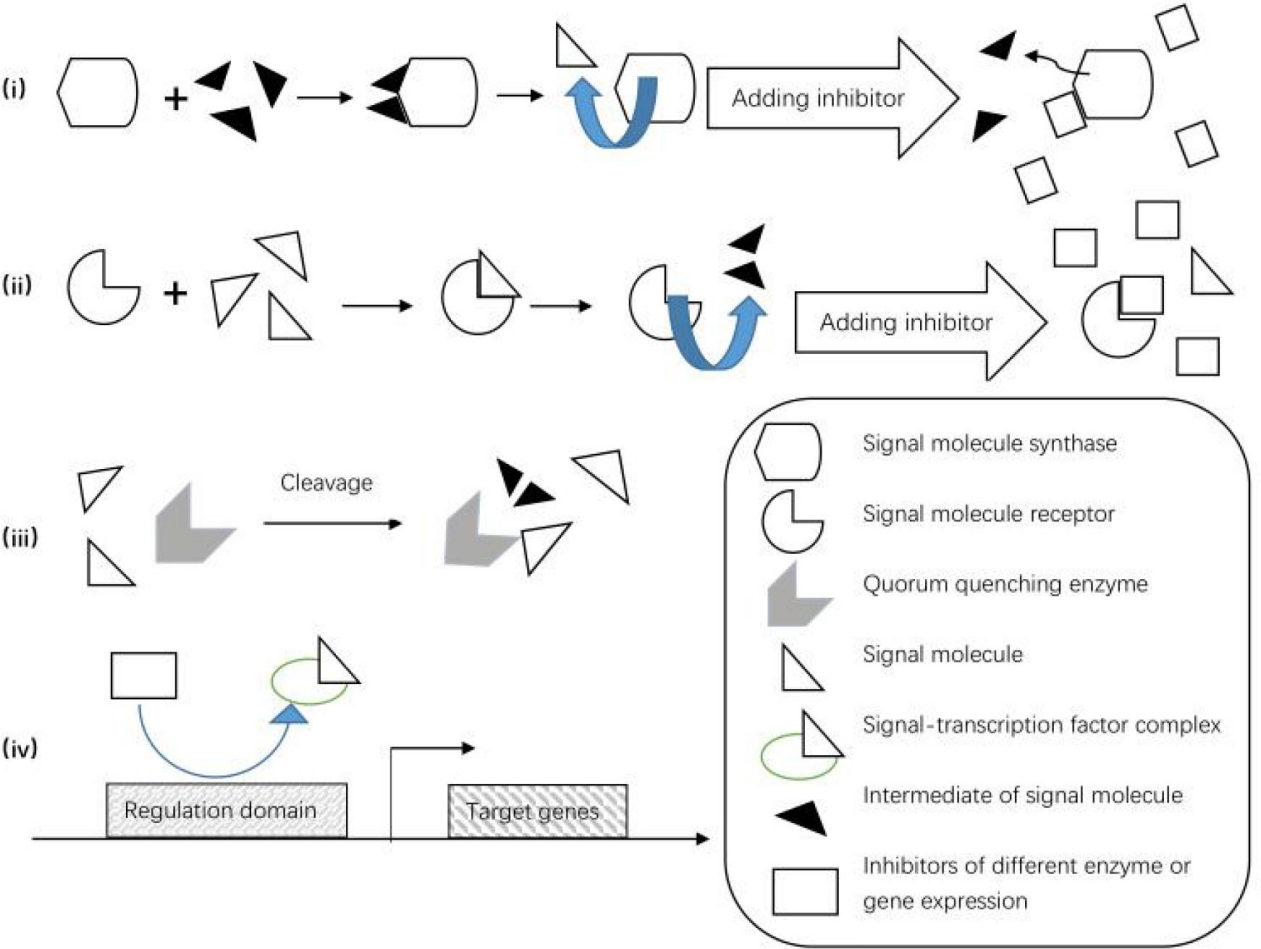
Mechanisms of quorum quenching, including signal production, perception and transmission through competitive inhibition or signal degradation, and inhibition of QS-related gene expression.

QQ mechanisms can be categorized into five main groups, as detailed below:

(i) Those which competitively inhibit the synthesis and perception of signaling molecules (Haruna et al., 2016; Rao, 2021). Chung et al. (2011) have characterized two previously unknown QS inhibitors against *Burkholderia glumae* in a library of acyl-HSL analogs. The first inhibitor, J8-C8, competitively binds to C8-HSL synthase TofI, occupying the binding site for the acyl chain of acyl-carrier protein; the second inhibitor, E9C3oxoC6, competitively binds TofR, the C8-HSL receptor (Chung et al., 2011); (ii) those which promote the degradation of signaling molecules (Haruna et al., 2016; Tom Defoirdt, 2011), including QQ enzymes and biocontrol QQ bacteria; (iii) those which interfere with the binding between transcription regulation factors and gene promoter sequences and down-regulate the expression of synthases and receptor genes (Haruna et al., 2016). It has been reported that mono-unsaturated fatty acids, palmitoleic acids (PoAs), and myristoleic acids (MoAs) down-regulate the expression of *abaR* in the QS system, thereby reducing the binding between AHLs and AbaR (Nicol et al., 2018). Above mentioned mechanisms of quorum quenching are vividly depicted in **Figure 2**.

Taken together, different QQ strategies share the same goal: impeding signaling molecules from binding with a specific receptor to control the gene expression in microbial metabolism, such that group behaviors regulated by the signaling mechanism can be hampered (Nazzaro et al., 2019).

### 4.2 The discovery and design of various QSIs

#### 4.2.1 QSIs from bacterial sources

Similarly, to the first report of QS in *V. fischeri* responsible for the bioluminescence, the QQ phenomenon was also first observed in marine organisms. An AHL homolog, a halogenated furanone produced by *Dellsea pulchra*, was found to be capable of inhibiting the swarming motility of *Serratia liquefaciens*, as well as the bioluminescence of *V. harveyi* and *V. fischeri* (Yu et al., 2018). Further studies have shown that halogenated furanones—one of the most common QSI families—target both AHL- and AI-2-mediated QS with distinct modes of action: the former reduces the stability or binding affinity of the LuxR regulator, while the latter inhibits the synthase, LuxS, through a covalent interaction (Bzdrenga et al., 2017). However, halogenated furanones are not the first choice for QQ strategies, due to their relatively poor stability.

#### 4.2.2 QSIs from plant extracts

Some natural chemical components, such as ethanolic extracts of Mango Seed Kernel Extract, Guava Leaf Extract, and ϵ-Polylysine (ϵ-PL), have been found to affect Methicillin-resistant *Staphylococcus aureus* (MRSA), in terms of motility and expression of δ-hemolysin activity, by interfering with its QS activity (Divyakolu et al., 2021). These results indicate that QSIs could be used as an adjunct to antibiotics, to reduce the development of drug resistance (Divyakolu et al., 2021). Tannins in pomegranates and berries are capable of reducing QS-regulated physiological activity by 40% and reducing the level of signaling molecules produced by the intestinal pathogen *Yersinia enterocolitica* (Zhao, 2020).

#### 4.2.3 Modern technology for QSI design

Computer-aided drug design has greatly improved the efficacy of exploring new QSIs. Among them, molecular docking is a widely-used method that can quickly and accurately simulate the docking of ligands with receptors, allowing for the determination of the molecular mechanism of substrate–enzyme interactions or efficiently obtaining target-specific compounds (e.g., those with the lowest binding affinity, binding energy, and docking score), including new types of QS inhibitors (Ewing et al., 2001; Bhatt et al., 2023). It has also been reported that the modification of natural AHLs—including introducing unsaturated bonds and altering the length of the carbon chain—is an effective and practical strategy for exploring chemical inhibitors of the LuxR family. For example, prolonging the carbon chain of C6-HSL to C10-HSL presented strong inhibition of CviR, the HSL receptor in *Chromobacterium violaceum* (Yan, 2018).

### 4.3 The family of AHL-quenching enzymes

**Figure 3:**
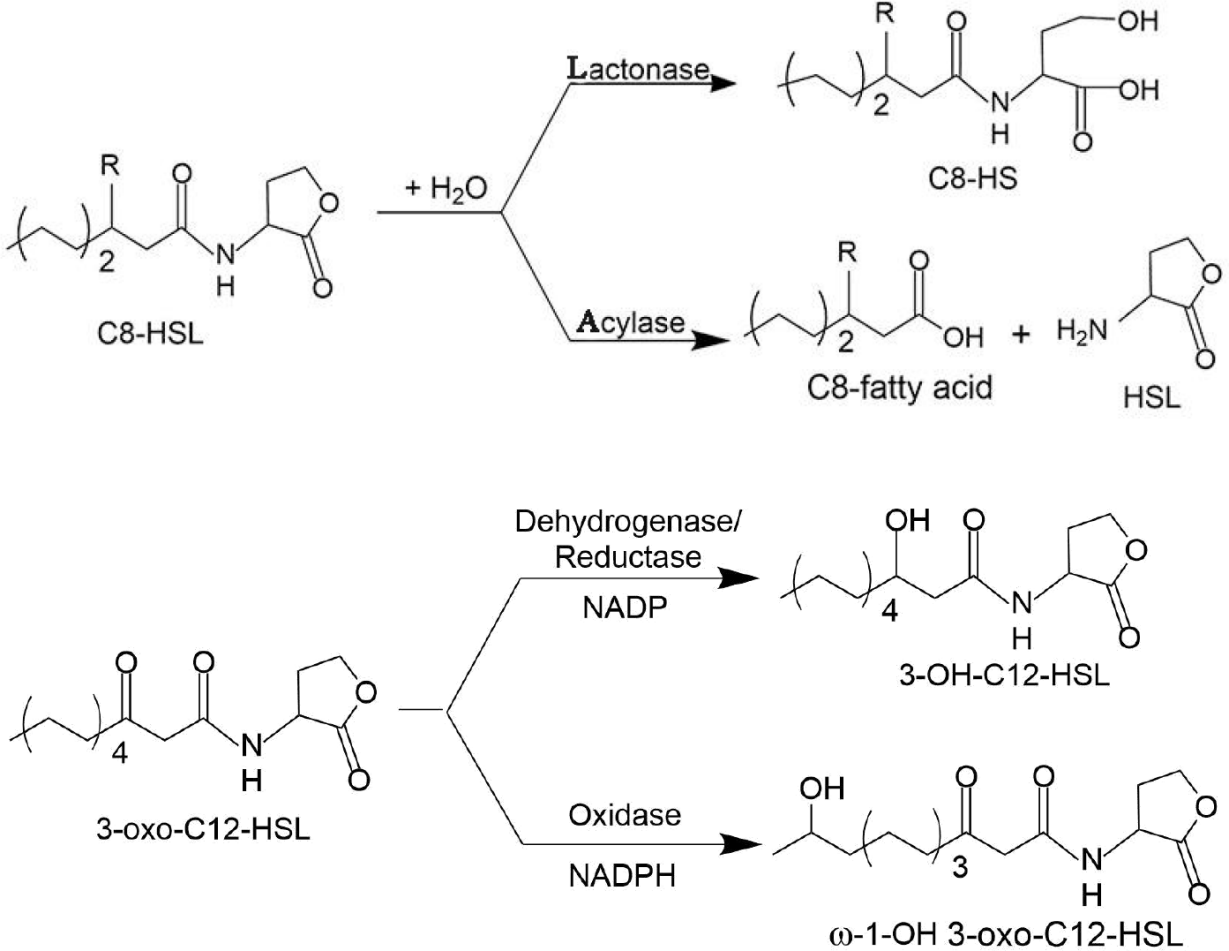
Mechanisms of AHL inactivation by lactonases, acylases, reductases, and oxidases: (1) AHL lactonases cleave homoserine lactone ring, generating acyl homoserine in reversible reaction; (2) AHL acylases catalyze the hydrolysis of AHL on amide bonds and generate fatty acid chains and homoserine lactone in an irreversible reaction; (3) NADP-dependent AHL reductases act on C3, reducing carbonyl groups to hydroxyl groups (Bijtenhoorn et al., 2011); (4) 3-Oxo-C12-HSL is oxidized at the ω-1, ω-2, ω-3, or even ω-4 and ω-5 carbons by NADPH-dependent oxidase (Chowdhary et al., 2007).

Since the first discovery of the AHL lactonase in *Bacillus cereus* (Dong, 2000) and the isolation of the AHL amidase from *Variovorax paradoxus* (Leadbetter and Greenberg, 2000) in 2000, the number of reports on AHL-degrading enzymes has steadily increased. According to their mode of action, AHL-inactivating enzymes can be divided into three categories (Fan et al., 2017), including lactonases, amidases, and oxidoreductases, which degrade or modify AHLs in three different ways (shown in **Figure 3**). In the following, bacterial AHL-quenching enzymes are described in detail, along with a brief introduction to enzymes from non-bacterial sources.

#### 4.3.1 AHL lactonases

**Figure 4:**
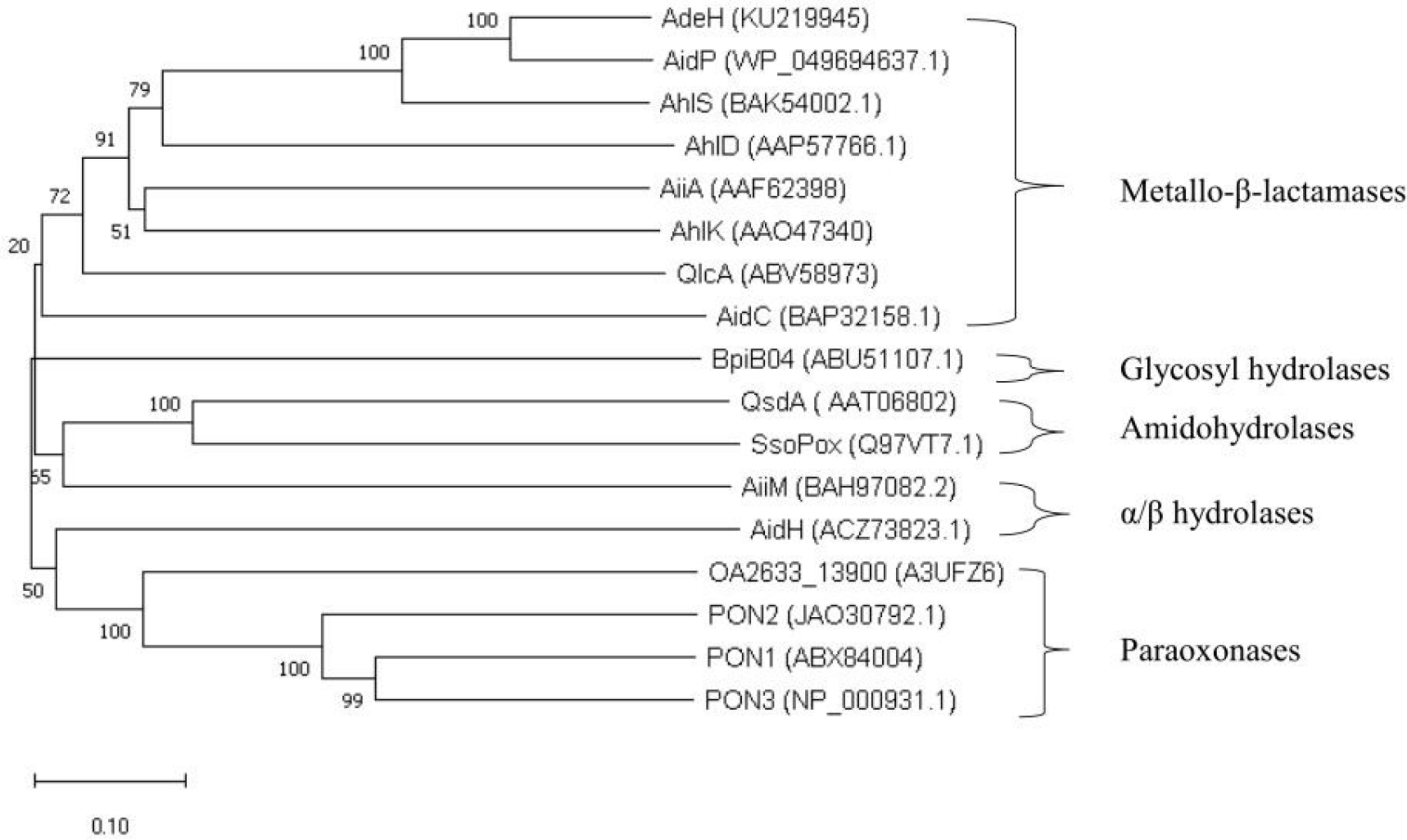
Phylogenetic tree of AHL lactonases. In this tree, the AHL-quenching enzymes are categorized into five families, and these quenching enzymes as follows: AidP (See-Too et al., 2017); AdeH (Garge and Nerurkar, 2016); AhlS (Morohoshi et al., 2012); AhlD (Park et al., 2003); AiiA (Dong, 2000); AhlK (Park et al., 2003); QlcA (Riaz et al., 2010); AidC (Wang et al., 2012); BpiB04 (Schipper et al., 2009); AiiM (Wang et al., 2010); AidH (Fan et al., 2020); QsdA (Hong et al., 2012); SsoPox (Park et al., 2006); OA2633_13900 (Oh et al., 2011) ; PON1, PON2, PON3 (Bar-Rogovsky et al., 2013). Using MEGA10.2.3, the 17 protein sequences were aligned by ClustalW, and the tree was constructed according to the neighbor-joining method.

To the best of our knowledge, most of the AHL-degrading enzymes are lactonases. These can be categorized into four groups (Bzdrenga et al., 2017; Fan et al., 2017; Chang et al., 2019): Metallo-β-lactamases, paraoxonases, α/β hydrolase lactonases, and phosphotriesterase-like lactonases. Some categorizations further include amidohydrolases and glycosyl hydrolases. The phylogenetic relationships between AHL lactonases are detailed in **Figure 4**.

1. Metallo-β-lactamases have a highly-conserved Zn^2+^ binding domain HXHXDH, where Zn^2+^ is necessary for their catalytic activity (Rao, 2021). Among AHL lactonases, metallo-β-lactamases are the most thoroughly studied ones, including AiiA from *Bacillus* sp. 240B1 (Dong, 2000), and AttM and AiiB from *Agrobacterium tumefaciens* C58 (Rao, 2021).
2. Paraoxonases are a group of enzymes (PON1, PON2, and PON3), which are highly conserved in vertebrates and, in particular, in mammals. PON2 efficiently inactivates AHLs and exhibits arylesterase activity (Draganov Di, 2005). Purified PON1 and PON3 show lower catalytic activity toward AHLs, but act on a wide range of substrates, including organophosphates, arylesters, gamma-lactones, and delta-lactones (Draganov Di, 2005). The heterogeneous expression of human PON1 in *Drosophila melanogaster* reduced OC12-HSL-mediated *P. aeruginosa* virulence, down-regulated the superoxide anion level, and modified the gut microbiota composition (Stoltz et al., 2008).
3. α/β hydrolase lactonases share a conserved nucleophile–histidine–acid catalytic trimer domain, which consists of a nucleophile chemical structure (G-X-Nuc-X-G), one histidine, and one acidic amino acid (Asp or Glu). AidH isolated from *Ochrobactrum* sp. T63 (Mei et al., 2010), AiiM from *Microbacterium testaceum* StLB037 (Wang et al., 2010), and JydB from *Rhodococcus* sp. BH4 belongs to this family (Rao, 2021).
4. Phosphotriesterase-like lactonases (PLLs) are members of the amidohydrolase family (Rao, 2021), with a relatively wide range of substrates. Some PLLs are called paraoxonases, due to their catalytic activity for organophosphorus paraoxon (Rao, 2021); while some PLLs exhibit lower phosphotriesterase activity, but can proficiently hydrolyze different lactones (Afriat et al., 2006). The reported PLLs include QsdA isolated from *Rhodococcus erythropolis* (Uroz et al., 2008), GKL from *Geobacillus kaustophilus* (Chow et al., 2010), and GsP from *G. stearothermophilus* (Hawwa et al., 2009).

#### 4.3.2 AHL acylases

Shortly after the discovery of the first QQ lactonase AiiA, a strain of *Variovorax paradoxus* was isolated, which hydrolyses the AHLs into homoserine lactone and corresponding fatty acids, utilizing them as nitrogen source and energy source (Fan et al., 2017). Except that a minority of acylases belong to the α/β-hydrolase family (Fan et al., 2017), most QQ acylases can be categorized into the N-terminal nucleophile (Ntn) hydrolase superfamily (Fan et al., 2017; Liu et al., 2017; Morohoshi et al., 2017), which can be divided into three groups: The aculeacin A family, penicillin G acylase family, and amidase family (Yu et al., 2018). Some characterized AHL acylases categorized by protein families are listed in **Table 1**.

**Table 1:** Reported AHL acylases isolated from QQ bacterial strains.

QQ enzymes	Protein families	Bacterial strains	References
AiiO	α/β-hydrolase	*Ochrobactrum* sp. A44	(Czajkowski et al., 2011)
ND	α/β-hydrolase	*Delftia* sp. VM4	(Maisuria and Nerurkar, 2015)
E101G/R230C GKL mutant	Phosphotriesterase-like lactonase	ND	(Xue et al., 2013)
AibP	N-terminal nucleophile (Ntn) hydrolase	*Brucella melitensis* 16M	(Terwagne et al., 2013)
QuiP	Ntn hydrolases	*Pseudomonas aeruginosa* PAO1	(Huang et al., 2006)
QuiP	Ntn-hydrolase	*Pseudomonas aeruginosa strain* QSP01	(Khalid et al., 2022)
Slac1, Slac2	Ntn-hydrolase	*Shewanella loihica*-PV4	(Philem et al., 2020)
PvdQ	Ntn-hydrolase	*Pseudomonas putida* QQ3	(Khalid et al., 2022)
PF2571	Ntn-hydrolase	*Pseudomonas fluorescens* PF08	(Wang D et al., 2022)
HacA (Psyr_1971)	Ntn hydrolase	*Pseudomonas syringae* pv*. syringae* B728a	(Shepherd and Lindow, 2009)
HacB (Psyr_4858)	Ntn hydrolase	*Pseudomonas syringae* pv. *syringae* B728a	(Shepherd and Lindow, 2009)
Aac	Aculeacin A (Ntn hydrolase)	*Ralstonia solanacearum* GMI1000	(Chen et al., 2009)
Aac (SO0918)	Aculeacin A(Ntn hydrolase)	*Shewanella oneidensis* MR-1	(Morohoshi et al., 2008)
AiiD	Aculeacin A(Ntn hydrolase)	*Ralstonia* sp. XJ12B	(Huang et al., 2003)
AhlM	Penicillin G (Ntn hydrolase)	*Streptomyces* sp. M664	(Lin et al., 2003)
GqqA	ND	*Komagataeibacter europaeus* CECT 8546	(Werner et al., 2021)
ND	ND	*Rhodococcus pyridinivorans* XN-36	(Zhou et al., 2022)
AiiC	ND	*Anabaena* sp. PCC 7120	(Huang et al., 2003)
ND	ND	*Roseomonas* sp. TAS13	(Nasuno et al., 2017)

ND, unknown.

To date, AHL acylases have been less-reported than AHL lactonases, likely because their larger molecular weight presents more difficulties for purification. AHL acylase specializes in degrading AHLs with a long side chain, and is poor in terms of catalyzing the degradation of AHLs with a short side chain (Yu et al., 2018).

#### 4.3.3 AHL oxidoreductases

AHL oxidoreductases, which can inactivate AHLs by oxidation or reduction, have been rarely reported. Unlike lactonases and acylases, the functional mechanism of AHL oxidoreductases varies from one to another, and may function on other molecules rather than QS signals (Yu et al., 2018). For example, CYP102A1 from *Bacillus megaterium* is a cytochrome P450 that can oxidize both saturated fatty acids and fatty acyl amino acids on ω-1, ω-2, and ω-3 carbons, with a preference for long fatty acid chain (Chowdhary et al., 2007). The activity of oxidized AHL dramatically declines, but remains at a low level (Fan et al., 2017). In the second case, *bpiB09* isolated from a soil metagenome has been characterized as an NADP-dependent reductase, capable of inactivating 3-oxo-C12-HSL by reducing the C3 carboxyl group to a hydroxyl group. The expression of bpiB09 in *P. aeruginosa* down-regulates the expression of QS-regulated virulence genes and reduces the paralysis of *Caenorhabditis elegans* (Bijtenhoorn et al., 2011).

#### 4.3.4 AHL-degrading enzymes in non-bacterial organisms

Other than bacteria, QQ enzymes have also been detected in other micro-organisms, and even in mammals. An AHL-degrading enzyme has been purified from *Rhodosporidium toruloides*, which was found to be tolerant to 2 mol/L NaCl or above, broadening the original scope of QQ enzymes in fungi (Luo, 2010). In a study of mammalian serum paraoxonases (PONs) related to bacterial homoserine lactonases (Bar-Rogovsky et al., 2013), it has been found that, similar to characterized bacterial PONX_OCCAL, mammalian ancestor PONs are capable of efficiently hydrolyzing *N*-acyl homoserine lactones, revealing the homology of PONs from mammals and bacteria.

### 4.4 Physical degradation of QS signals

In addition to digestion by QQ enzymes or inhibition by QSIs, QS signals can also be quenched under certain physical conditions. A featured case in point is the lactonolysis of AHL compounds—a phenomenon that should also affect all lactone derivatives—which can occur spontaneously in aqueous solutions. It is strongly favored at high temperatures and under alkaline pH, and can be reversed under acidic pH solutions. Under laboratory conditions, short-chain AHLs are more prone to degradation than long-chain AHLs, and the half-life of *N*-hexanoyl-homoserine lactone (C6-HSL) varies from over 21 days (pH 5.5, 4 °C) to less than 30 min (pH 8.5, 37 °C) (Grandclément et al., 2016).

Moreover, it has been shown that photocatalytic technology can effectively interfere with QS systems. Biofilm development was substantially delayed by TiO_2_ under UV irradiation, although no obvious cytotoxicity to cell growth was observed. The generated reactive oxygen species (ROS) were found to be capable of quenching the AI-2 secreted by *E. coli* K12 and suppressing the expression of two biofilm formation-related genes in *E. coli* K12 (*motA* and *rcsB*). Photogenerated ·OH and O2^•−^ radicals are believed to non-selectively target various kinds of organic pollutants, such as AHLs, as well as membrane surface and bacterial cells (Xiao et al., 2016).

## 5 The application of QQ strategies

Considering the obvious disadvantages of traditional strategies against pathogens, including antibiotics, QQ enzymes have presented an attractive and profound potential for inhibiting community adherence and virulent attack of pathogens using QS systems. Considering the universality of QS-mediated pathogenic activity, QQ strategies are applicable in various fields to combat pathogens, including agriculture, aquaculture, and waste treatment. Some traditional fields in which QQ strategies have shown promise for solving troublesome issues are detailed below. Experimental and practical applications of different QS inhibitors in these fields are also discussed, as summarized in **Table 2**.

**Table 2:** Quorum quenchers isolated from various sources and their applications.

Fields	Applications and its effect	Quorum quenchers	Representative substrate of quenchers
Agriculture	Reducing its pathogenicity of *Pectobacterium carotovorum* on potato slices	AiiA from *B. thuringiensis* (Dong et al., 2004)	*N*-(3-oxo-hexanoyl)-L-homoserine lactone (OHHL)
	Develop tobacco and potato transgenic lines with increased tolerance to *Erwinia carotovorum*	AiiA from *Bacillus* sp. (Zhang et al., 2001)	OHHL
	Attenuate soft rot caused by *Dickeya zeae* EC1	*Pseudomonas nitroreducens* strain W-7 (Zhang et al., 2021)	*N*-oxododecanoyl-L-homoserine lactone(OdDHL)
	ND	AciJ from *Acinetobacter*. sp 3-59 (Zhang et al., 2018)	acyl homoserine lactones (AHLs)
	Attenuate tobacco bacterial wilt disease caused by *Ralstonia pseudosolanacearum*	AiiA from *B. cereus* strain B15 (Zheng et al., 2021)	AHLs
	Reduce the severity of black rot disease in radishes and Chinese cabbage caused by *Xcc*	*Burkholderia anthina* strain HN-8 (Ye et al., 2020a)	cis-11-methyl-2-dodecylene acid(DSF)
	Expression of *fadT* in *Xcc* attenuating the pathogenicity in host plants	fadT from *Cupriavidus pinatubonensis* HN-2 (Xu et al., 2021)	DSF
	Expression of *fadY* in *Xcc* attenuating the pathogenicity in host plants	fadY from *Acinetobacter lactucae* QL-1 (Ye et al., 2020c)	DSF
Aquaculture	Oral administration reduces *Aeromonas hydrophila* level	Goldfish basal diet prepared by *Bacillus* sp.QSI-1 (Zhou et al., 2016)	Various AHLs
	Co-injection of purified YtnP and *A. hydrophila* decrease mortality of goldfish	YtnP from *B. licheniformis* T-1 (Peng et al., 2021)	*N*-hexanoyl-homoserine-lactone (C6-HSL)
	Oral administration decrease *A. hydrophila* infection in zebrafish	AiiA_AI96_ from *Bacillus* sp. strain AI96 (Cao et al., 2012)	*N*-(3-oxo-octanoyl)-L-homoserine lactone (3-oxo-C8-HSL)
	Reduce colonization of *Vibrio parahaemolyticus* of Indian white shrimp and its mortality rate	AiiA from *B. licheniformis* DAHB1 (Vinoj et al., 2014)	C6-HSL
Antifouling	Reduce biofilm surface coverage by 97% on reverse osmosis membrane	Vanillin (a natural QQ compound) (Kappachery et al., 2010)	AHL (predicted)
	Down-regulate the concentration of AI-2 and EPS, reducing microbial attachment to glass and polypropylene surfaces	D-tyrosine (Xu and Liu, 2011)	autoinducer-2 (AI-2)
	Prohibit EPS secretion and biofilm formation, increase the maintenance of its initial flux by 30% after 38 h operation	Acylase-immobilized nanofiltration membrane (Kim et al., 2011)	ND
	Delay TMP increase rate and mitigate transmembrane pressure build-up in membrane bioreactor	*Rhodococcus* sp. BH4 -entrapping beads (Lee et al., 2016)	*N*-octanoyl-L-homoserine lactone (C8-HSL)

ND, unknown.

### 5.1 Virulence attenuation of agricultural pathogens

#### 5.1.1 Necessities of applying quorum quenchers

Bacterial plant disease is one of the most important natural disasters in crops, causing serious economic losses. It not only leads to decreased crop yield but also seriously threatens the quality and safety of agricultural products (Alexander, 2010). Pesticides, traditionally used and widely applied agents to fight against plant pathogens (Morillo and Villaverde, 2017), cause serious soil and water pollution when used excessively (Carvalho, 2017; Li et al., 2022; Zhang et al., 2022). Therefore, the QS system provides a new target for the inhibition of pathogen virulence and avoiding traditional resistance, and has consequently drawn significant attention. It has been found that many bacterial pathogenic behaviors are under strict regulation of the QS system, where some typical examples are listed below.

On one hand, plant pathogens can form biofilms at the leaf, rhizosphere, and vascular bundle levels, to keep the cells aggregated for plant infection. For example, *Xanthomonas campestris* pv. *campestris* (*Xcc*), *Pectobacterium carotovorum* subsp*. brasiliense*, and *Clavibacter michiganensis* subsp. *sepedonicus*, form biofilms in plant vascular tissues, blocking and destroying infected tissues (Song et al., 2017).

On the other hand, plant pathogens can secret hydrolases for infection. *Pectobacterium carotovorum* subsp. *carotovora*, a pathogen that synthesizes extracellular hydrolases such as gumase, galacturonase, and pectinase, destroys plant cell walls and causes soft rot disease (Lee et al., 2013). The production of these extracellular hydrolases is strictly regulated by the ExpI/ExpR system (Andersson et al., 2000). Meanwhile, the exopolysaccharide production of *Pantoea stewartii* ssp. *Stewartii* is regulated by the EsaI/EsaR system (Koutsoudis et al., 2006).

#### 5.1.2 The identification and characterization of biocontrol strains

To attenuate the pathogenicity of plant pathogens, many AHL or DSF-degrading strains have been isolated and characterized.

In 2004, *B. thuringiensis*, a traditional biological pesticide producing endotoxins lethal to moths, butterflies, and mosquitoes (Palma et al., 2014), was shown to produce a lactonase (called AiiA) which inactivates the AHL produced by *Pectobacterium carotovorum*, thereby reducing its pathogenicity on potato slices (Dong et al., 2004). To improve the catalytic efficacy of AiiA, Zhang et al. (2007) fused AiiA with a secretive protein to enhance its dispersion in the environment, resulting in increased tolerance to *P. carotovorum* in potato. Enormous efforts have been made to screen and identify QQ strains. For example, *Pseudomonas nitroreducens* strain W-7, a highly efficient and wide-range AHL-degrading strain, has been isolated from activated sludge samples. It was capable of degrading 0.2 mmol/L of OdDHL within 48 h, and could substantially attenuate the soft rot caused by *Dickeya zeae* EC1 (Zhang et al., 2021). In another case, *Acinetobacter*. sp 3-59, showed strong intracellular AHL-degrading activity, and the AHL-degrading gene *aciJ* exhibited above 90% similarity to the mono-oxygenase of *Acinetobacter.* sp (Zhang et al., 2018). In the research of Zheng et al. (2021), to screen out aiiA-housing bacteria against *Ralstonia pseudosolanacearum* from 253 biocontrol strains, degenerate primers were designed and aiiA was amplified by PCR. After 12 strains were obtained, *B. cereus* strain B15 was selected. Its biocontrol effect on bacterial wilt of tobacco was 68.33% after 14 days of inoculation, higher than other strains (0–60.00%) and chemical treatment (Zheng et al., 2021).

In recent years, some biocontrol strains capable of degrading DSF have been isolated, including *Burkholderia anthina* strain HN-8 (Ye et al., 2020a), *Acinetobacter lactucae* strain QL-1 (Ye et al., 2019), *Cupriavidus pinatubonensis* strain HN-2 (Xu et al., 2021), and *Burkholderia* sp. F25 (Yu et al., 2022). *Burkholderia anthina* HN-8 exhibited superb DSF degradation activity and completely degraded 2 mM DSF within 48 h. Further research on this strain presented the first evidence of a bacterium having a metabolic pathway for the complete degradation and metabolism of DSF, revealing that DSF could be inactivated by oxidation–reduction (Ye et al., 2020b). For strains QL-1 and HN-2, *fadY* encoding fatty acyl-CoA synthase and *fadT* encoding acyl-CoA dehydrogenase have been characterized as key DSF-degrading genes through whole-genome sequencing and comparative genomics studies (Ye et al., 2020c; Xu et al., 2021). Moreover, the expression of *fadT* and *fadY* in *Xcc* resulted in significantly decreased pathogenicity in host plants, such as Chinese cabbage and radish (Ye et al., 2020c; Xu et al., 2021).

#### 5.1.3 The development of QQ enzyme-producing transgenic plants

Some plants have been genetically modified with QQ genes, such as those derived from *Bacillus* spp. or *A. tumefaciens*, which allow them to produce lactonases. The first transgenic lines, *Bacillus aiiA*-transformed tobacco, and potato lines, were reported in 2001 (Zhang et al., 2001). They presented an increased tolerance to *P. carotovorum*, with symptoms only appearing after inoculation with very high bacterial concentrations (Zhang et al., 2001). In transgenic tobacco expressing attM, a QQ gene cloned from *Agrobacterium tumefaciens*, wilt symptoms and mortality were dramatically decreased (Niu et al., 2012).

#### 5.1.4 Limitations of studies considering agricultural applications

First, QQ enzyme agents protecting plants from bacterial infections are an attractive alternative to genetically modified plants but are impaired by the poor stability of the enzymes. To deal with this issue, the development of environmentally stable and chemically resistant enzymes is crucial.

Second, the impacts of QQ enzymes on beneficial or symbiotic bacteria must be further considered. The situation in the field is different from that in the laboratory, and further research is needed to balance its drawbacks against its beneficial impact.

Third, we should extend the sources of QQ agents. In rhizosphere soils, some biocontrol bacteria can release interspecies signaling molecules and interfere with the QS signaling pathway in pathogenic bacteria, causing the reduction of their pathogenic characteristics, thus shedding light on the development of new control strategies (Haruna et al., 2016).

### 5.2 Application of QQ strategies in aquaculture

Bacterial infections severely threaten aquaculture. Traditional methods, including probiotics, bacteriophage therapies, immunostimulants, and vaccines, fail to effectively control bacterial diseases (Bzdrenga et al., 2017). Some QQ strains have been identified and characterized for the attenuation of virulence in fish pathogens (Bzdrenga et al., 2017).

#### 5.2.1 Fish intestinal flora and fish health

Bacterial enteritis is probably the most common intestinal disease in freshwater fish (Macpherson et al., 2012). *Aeromonas hydrophila*, a Gram-negative bacterium, is generally considered to be a significant pathogen causing fish enteritis and bacterial sepsis (Cascon et al., 2000). It is prevalent in many areas, and leads to high fish mortality rates, seriously restricting the development of aquaculture in China (Li, 2016). When infected by *A. hydrophila*, the proportion of the dominant bacteria (*Pachychychia*, *Proteobacteria*, and *Bacillus*) in the intestinal flora of grass carp decreases, while the proportion of *Clostridium* increases (Li, 2016), providing strong evidence that the pathogenicity is correlated with intestinal flora change in fish.

#### 5.2.2 Virulence attenuation of aquacultural pathogens

QQ enzymes and QQ bacteria have been reported to attenuate the pathogenicity of *A. hydrophila*. It has also been acknowledged that QQ enzymes have a more rapid effect in regulating microbial flora than QQ bacteria (Zhao, 2020).

It has been confirmed that oral administration of QQ bacteria agents or purified QQ enzymes provides an effective means to attenuate *A. hydrophila* infection. Purified lactonase AiiA_AI96_ from *Bacillus* sp. strain AI96 has been shown to decrease infection in zebrafish, which was the first study to report that the oral administration of an AHL lactonase can effectively control *A. hydrophila* (Cao et al., 2012). AiiA_AI96_, categorized as a member of the metallo-β-lactamase superfamily, is resistant to protease and carp intestinal juice digestion, and maintains stability at 70 °C and pH 8.0 for at least 1 h (Cao et al., 2012). Zhou et al. (2016) fed *Carassius auratus* (goldfish) with a QQ bacteria, *Bacillus* sp. QSI-1, and observed an increase in Proteobacteria and decrease in *Clostridium*, demonstrating that QQ bacteria modify the fish gut microbiota. At the same time, the percentage of *A. hydrophila* decreased significantly, which strongly suggests the potential of QQ probiotics to control aquacultural bacterial diseases. According to a recent study, after AiiO-AIO6 was added to zebrafish feed, the PCR analysis showed that the transcription of many nutrient transporters and growth-related genes (e.g., the peptide transporter *Pept1a*, glucose transporter *GLUT2*, and growth hormone receptor *GHra*) were all significantly up-regulated, when compared with the control (Das et al., 2022).

Moreover, a report has shown that the injection of AiiA from *B. licheniformis* DAHB1 into the abdominal cavity successfully reduced the colonization of *V. parahaemolyticus* in Indian white shrimp, as well as the infection and mortality rates in shrimp (Vinoj et al., 2014). AiiA produced by *B. licheniformis* inactivated a wide range of AHL substrates and could tolerate the acidic environment in the shrimp intestine (Romero, 2012). Another report has shown that co-injection of purified YtnP and *A. hydrophila* altered the richness of intestinal flora and decreased mortality by more than 50% at 96 h, through attenuating the pathogenicity of *A. hydrophila* (Peng et al., 2021).

Taken together, these results provide valuable insights into the QQ enzymes and bacteria that modulate the microbiota structure, thus attenuating the virulence of pathogens in fish and shrimp, as well as even boosting their growth by regulating gene expression.

### 5.3 Biofouling mitigation of membrane bioreactors

Membrane bioreactors (MBRs) and anaerobic membrane bioreactors (AnMBRs), which combine a classic bioreactor system with a membrane filtration step, have been widely used in wastewater treatment for the bacterial cleaning of soluble pollutants through the retention of micro-organisms and solid particles (Drews, 2010; Anjum et al., 2021). As a major concern encountered in such systems (Ni and Wang, 2019), membrane fouling (particularly, biofouling) is mainly due to the attachment and proliferation of microbes on the membrane surface, resulting in biofilm formation (Ni et al., 2021). Membrane fouling causes lower membrane filterability, increases energy consumption, and calls for the frequent cleaning or replacement of membrane modules (Syafiuddin et al., 2021). In biofouling control of MBRs or AnMBRs systems, transmembrane pressure (TMP) is one of the indicators that can be used to evaluate membrane permeability (Anjum et al., 2021; Syafiuddin et al., 2021). The increase in TMP at different profiles could be due to pore blockage and EPS accumulation on the surface of the membrane (Syafiuddin et al., 2021). When the TMP increases, conventional cleaning strategies fail to perform well: physical cleaning presents an incapability to remove all fouling layers, while chemical cleaning potentially induces chemical resistance of the micro-organisms, thus reducing the biofouling control efficiency (Syafiuddin et al., 2021).

Therefore, current strategies focus on using biological approaches which aim to avoid the drawbacks associated with physical and chemical approaches (Shah and Choo, 2020). Since Yeon et al. demonstrated the relationship between QS signaling and membrane fouling (Yeon et al., 2009a), many scholars have attempted to apply QQ strategies to membrane filtration, and have investigated the potential of various QQ agents, such as QQ compounds, QQ enzymes, QQ bacteria, and so on (Ni and Wang, 2019). It is believed that QQ strategies have the potential to not only handle MBR biofilm formation but also exert no adverse effects on the capability of MBRs, in terms of organic and nutrient removal (Xiao et al., 2018), thus benefiting the long-term stability of MBRs and their application at wider scales.

#### 5.3.1 QQ enzymes and QQ bacteria for biofouling control

It has been reported that immobilizing QQ enzymes provides an effective strategy for improving the stability and prolonging the lifespan of the enzymes. Kim et al. (2011) have found that an acylase-immobilized QQ membrane prohibited the formation of a mature biofilm, due to the reduced secretion of extracellular polymeric substances. This membrane maintained more than 90% of its initial enzyme activity for more than 20 iterative cycles of the reaction and washing procedure, as well as more than 90% of its initial flux after 38 h of operation. Yeon et al. (2009b) have reported a magnetic enzyme carrier (MEC) prepared by immobilizing acylase on magnetic particles. This MEC not only efficiently alleviates membrane biofouling but also presents great advantages over free enzymes in terms of stability, with no activity decrease under both continuous shaking for 14 days and 29 iterative cycles of reuse.

The isolation and application of QQ strains have also been frequently reported in the field of biofouling control. Noori et al. (2022) have isolated two QQ strains of *Bacillus* spp. from the activated sludge used to treat industrial wastewater containing two toxic pollutants: tetramethylammonium hydroxide (TMAH) and 1-methyl-2-pyrrolidinone. Co-culturing *Bacillus* spp. with *P. aeruginosa* PAO1 or activated sludge significantly reduced the biofilm formation of PAO1 and mixed communities in activated sludge.

Various carriers have been applied to load the QQ bacteria and improve the degradation rate of signaling molecules. Lee et al. (2020) have developed a mesoporous silica medium entrapping *Rhodococcus* sp. BH4, a dominant QQ bacteria strain employed to control MBR biofouling. The degradation rate of C8-HSL using the live BH_4_-entrapped medium was 80% higher than that of the control group (silica medium containing dead BH4). It is believed that the live BH4 medium can remove AHLs through both adsorption (mesoporous hydrophobic structure) and enzymatic decomposition (QQ) (Syafiuddin et al., 2021). Another carrier, QQ bacterial entrapping beads (QQ-beads) have also been commonly used as carriers of QQ strains for (An)MBRs biofouling control. QQ-beads are often prepared as mixtures of re-suspended QQ bacteria and polymer solutions (e.g., sodium alginate and polyvinyl alcohol). The beads can be further used directly or coated with a polymer before application in biofouling control. It has been suggested that a polymeric coating can improve the effective lifetime of beads, but likely slows the release of QQ enzymes into the MBR system (Syafiuddin et al., 2021). The addition of QQ-beads into a conventional MBR substantially affected the EPS concentrations, as well as microbial flora size in the mixed liquor, thus reducing TMP build-up (Lee et al., 2016). Other than mesoporous silica and beads, other forms of QQ agents have also been applied in MBR biofouling control. Shu et al. (2022) investigated the direct injection of different doses of unentrapped QQ strains (*Rhodococcus* sp. BH_4_) into a probiotic QQ MBR, which demonstrated effective biofouling mitigation in diverse MBR phases.

Other improvements can be made to maintain the good performance of MBRs: Khan et al. (2019) have reported the synergetic effects of QQ bacteria (*Rhodococcus* sp. BH_4_) and the electric field in a submerged membrane electro-bioreactor (SMEBR), and showed significant improvements, compared to conventional submerged MBR, in terms of delaying TMP increase, while maintaining higher removal of COD, ammonium-N (NH4^+^-N), and phosphorus-P (PO_4_^3-^).

In long-term continuous MBR operations, TMP is seldom used as an indicator, except in the study of Dong et al. (2022). In this study, a QQ system exploiting two QQ bacteria (*Serratia* sp. Z4 and *Klebsiella* sp. Q2) and γ-caprolactone (GCL) was shown to delay the TMP increase by half during 40 days of operation in a continuous MBR with low effluent chemical oxygen demand and ammonium nitrogen (Dong et al., 2022). This highlighted the effectiveness of QQ bacteria in mitigating biofouling through the stimulation of GCL in the long-term operation of MBR.

#### 5.3.2 Other QQ strategies in biofouling control

Multiple natural compounds can play the role of quorum quenchers in mitigating biofouling. Kappachery et al. (2010) have applied vanillin in bioreactors, and found that it can inhibit biofilm growth on the reverse osmosis membrane, with an inhibition rate of 97%. Xu and Liu (2011) observed that D-tyrosine dramatically down-regulated the concentration of AI-2 and extracellular polysaccharides, thus reducing microbial attachment to glass and polypropylene surfaces. A polyphenolic extract from Rosa rugose (Zhang et al., 2014), piper beetle extract (Siddiqui et al., 2012), and carvacrol (Gutierrez-Pacheco et al., 2018) have also been found to interfere with the bacterial QS system and attenuate biofilm formation on the membrane surface.

Physical degradation is also viewed as an effective strategy for quenching signaling molecules. In a photocatalytic membrane reactor system, the reactive oxygen species (ROS) generated by UV-excited TiO_2_ could oxidize the membrane surface foulants and quench AHLs. Additionally, this *in-situ* membrane cleaning strategy enhanced chemical oxygen demand (COD) removal, and almost complete disinfection of the effluent was realized in the photocatalytic QQ system. Treatment consisting of 45 min of UV irradiation (15 W) on QQ beads led to 95% degradation of AHL. This photocatalytic strategy can effectively mitigate the TMP from 30 kDa to 5 kDa in 4 h (Mehmood et al., 2021), showing its great potential for *in-situ* membrane cleaning.

#### 5.3.3 Limitations of QQ strategies in antifouling applications

First of all, the cost of practical applications is the basic factor limiting the application of QQ strategies. For example, extracting plant-sourced QQ compounds and the following purification requires sophisticated procedures, which are considered to be economically unfriendly, restricting its application (Ni and Wang, 2019).

Second, considering the sophistication of micro-organism communities and the diversity of QS signals, we can hardly rely on a single quencher to effectively mitigate biofilm formation and accumulation (Ni and Wang, 2019); for example, the QQ consortium developed by Xiao et al. (2018) provided a selective pressure on the biocake not only by preventing Gram-negative bacteria but also enriching Gram-positive bacteria, due to its capability to degrade AHLs but not AIPs. The sophisticated mechanisms of QS still need to be uncovered, and the combined effect of multiple quenchers deserves further investigation.

Third, how to precisely limit the QQ effect on the membrane surface should be paid more attention to. As far as MBR is concerned, most QQ strategies have a durative effect, and the long-term inhibition of biofilm formation may harm normal bacterial growth, thus hampering the water purification efficacy.

### 5.4 Attenuating virulence of human pathogenic bacteria

Many studies have been conducted to verify the effect of diverse QQ agents on *P. aeruginosa*, a clinically noted human pathogen that regulates virulence *via* a complex QS system involving natural or synthetic compounds, QQ strains or enzymes, and even antibody targeting signal molecules (Defoirdt, 2018). Bernabè et al. (2022) identified GM-50 as the most active compound in a library of small phenolic derivatives. It prevents adhesion of PAO1 and inflammatory damage in the human A549 cell line and significantly reduces virulence factors in twenty *P. aeruginosa* clinical isolates from patients with respiratory tract infections.

Inhibition of the QS system is reported to attenuate bacterial antibiotic resistance and, as a consequence, its significantly reduced usage dose. Ivanova et al. (2022) clinically applied the antibiotic gentamicin with an acylase from *Aspergillus melleus* using an one-step ultrasound emulsification process and found that the generated hybrid gentamicin/acylase nanospheres showed 16-fold improved bactericidal activity toward *P. aeruginosa* compared with pure gentamicin. The nanohybrids also attenuated 97 ± 1.8% of the production of violacien (a virulence factor) in *Chromobacterium violaceum*.

In terms of the application of QQ agents in clinical treatment, the stability of QQ agents and their capacity to reach their targets are currently the major obstacles encountered in their use. New approaches of nanocarrier delivery are proven to be an effective tool, and this is supported by the effective delivery of niclosamide nanoparticles to inhibit *P. aeruginosa* QS at concentrations of 2.5−10 μM in aerosol form, providing a tool for the local treatment of *P. aeruginosa* lung infections as in the case of CF patients (Costabile et al., 2015). Another case in point is reported by Lu et al. (2015), in which QS signal-contained CAI-1 nanoparticles can diffuse across delivery barriers, such as mice intestinal mucus, and regulate *V. cholerae* QS responses much more effectively than free CAI-1.

When considering the clinical application of QQ agents, their influence on the host microbiota must be taken into account. Although QQ agents have long been considered to have fewer side effects toward nontarget organisms than conventional antibiotics, they still require further investigation on this aspect, especially for the broad spectrum QQ agents. QQ agents can interfere with the colonization of beneficial bacteria, diminishing their positive effects on the host.

## 6 Conclusions and future perspectives

Based on the quorum sensing mechanism, quorum quenching has been promoted as an efficient biological control strategy, with a promising future in agricultural, aquacultural, and waste management applications. Compared to traditional antibiotics, quorum quenchers generate much less selective pressure—and, thus, less resistance—serving as a potential substitute (or, at least, supplements) to traditional antibiotics. Among the various quorum quenchers, QQ enzymes exhibit the best efficacy and lowest cytotoxicity.

Regardless of their great advantages, several problems remain unsolved for their practical applications. First of all, it has been recognized that QQ enzymes exhibit better QQ efficacy; however, they present lower stability in catalytic activity, when compared to QQ bacteria. It is, therefore, of great significance that the stability of QQ enzymes is improved, to accommodate for application circumstances. Efforts have been dedicated to the isolation of robust enzymes from extreme environments or the immobilization of QQ bacteria and QQ enzymes. Moreover, the targeting and delivery of QQ enzymes or QQ molecules, as well as evaluation of their cytotoxicity or other side-effects at the population, organism, cellular, and sub-cellular levels, are worth consideration. Furthermore, other than disrupting AHL- or DSF-based QS mechanisms, the quest for enzymes targeting AI-2, AI-3, or even AIPs, is essential for uncovering the potential of inhibiting a wider panel of Gram-negative and Gram-positive pathogenic bacteria.

## Author contributions

SC, L-HZ, KB and JW conceived of the presented idea. XZ contributed to the writing and prepared the figures and tables. W-JC, KB, ZZ, YH, SC and JW participated in revising the manuscript. All authors contributed to the article and approved the submitted version.
